# The Public Servants’ Response When Facing Pandemic: The Role of Public Service Motivation, Accountability Pressure, and Emergency Response Capacity

**DOI:** 10.3390/healthcare9050529

**Published:** 2021-05-01

**Authors:** Yong Ye, Yang Liu, Xiaojun Zhang

**Affiliations:** 1 School of Economics and Management, Fuzhou University, Fuzhou 350108, China; yeyong920@fzu.edu.cn (Y.Y.); t19106@fzu.edu.cn (Y.L.); 2 Integrity and Governance Research Center, Fuzhou University, Fuzhou 350108, China; 3 Fujian Emergency Management Research Center, Fuzhou University, Fuzhou 350108, China

**Keywords:** public servant, public service motivation, accountability pressure, emergency response capacity, COVID-19

## Abstract

(1) Background: Public servants are regarded as guardians of the public interest, and their pandemic response played a vital role in controlling the spread of the epidemic. However, there is limited knowledge of the factors that influence public servants’ response (PSR) when facing pandemic prevention and control tasks. (2) Methods: Based on the theory of planned behavior (TPB), models were constructed and a regression method was employed with Chinese civil servant data to investigate how PSR is influenced by public service motivation (PSM), accountability pressure (AP), and emergency response capacity (ERC). (3) Results and discussion: PSM, AP, and ERC all have a positive effect on PSR, with AP having the greatest influence, followed by PSM and ERC. The effects of PSM, AP, and ERC on PSR have group heterogeneity, which had little effect on civil servants with very low levels of PSR and the greatest impact on civil servants with medium-level PSR. Job categories of civil servants also are a factor related to PSR; PSM and AP have the strongest effects on civil servants in professional technology, and ERC has the greatest influence on administrative law enforcement. Moreover, gender, administrative level, and leadership positions also have an impact on PSR. (4) Conclusions: Based on the factors of PSR, we found at least three important aspects that governments need to consider in encouraging PSR when facing a pandemic.

## 1. Introduction

Public servants have an obligation to help citizens realize their own interests and rights and to achieve the goals of the public, society, state, and nation [1]. The effectiveness of epidemic prevention and control of COVID-19 crucially depends on the effort and capacity of the millions of public sector workers from the front line to central administration [2]. Public servants adopt many measures to prevent and control the pandemic, including assisting health administrative departments and other relevant departments in collecting information, distributing and segregating personnel, and providing infectious disease prevention and control information to citizens [3]. The behaviors of public servants are critical in an epidemic. For the state, their behaviors are related to the (in)ability to deliver policies, achieve goals [4], and improve efficiency [5], as well as the public trust in the government [6]. Trust in the government was correlated with decisions to abide by public health policies, restrictions, and guidelines [7]. For instance, in Nigeria, the loss of public confidence and vaccination boycott led to a resurgence of polio cases in 2004, which spread to more than 12 neighboring countries [8]. In 2015, the outbreak of measles in Orange County, California, was associated with similar concerns, which were exacerbated by parents’ distrust of US public health agencies [9]. A lack of trust in public health officials may lead to negative effects on the utilization of health services [10]. For society, the behaviors of public servants have relevance to the public service quality [11] and the creation and promotion of public value [12].

However, there is a dilemma between self-protection and service provision when public servants face pandemic risk [13]. The pandemic has fundamentally changed the workplace, work tasks, and the demands of public servants, which may create a significant strain on public sector workers [14], risking burnout [15], sick leave [16], demotivation [17], and lower performance [2]. Meanwhile, demands outside the workplace have also changed. With nursery and school being closed, public servants need to balance work and duties even during working hours. Moreover, the crisis has created higher ambiguities [4]; influenced the discretions of public servants [18]; and reduced public servants’ compound knowledge of task-specific intelligence, scientific knowledge, and policy rules, which made them doubt about how to make the best professional judgments in encounters with families and citizens [13]. In the light of job demands–resources theory (JD-R theory), job demands are positively associated with burnout, whereas job resources are positively related to engagement [19]. During the pandemic, job demands of public servants have increased, while job resources, such as equipment and support from colleagues, have suffered. Thus, public servants may exhibit lower work engagement [20]. Surveys of public servants are considered to be a useful management diagnostic that can aid governments in improving staff management (see, e.g., (Office of Personnel Management (OPM), 2019) U.K. Cabinet Office. 2018). The Bureaucracy Lab of the World Bank and its Global Survey of Public Servants Consortium carried out a COVID-19 Survey of Public Servants to identify the challenges and problems posed to public servants by COVID-19, as well as their response. However, the survey failed to analyze in depth the public servants’ responses during the pandemic. The theoretical research about epidemic prevention behaviors of public servants lags behind the practice.

In summary, the preventative behaviors of public servants are significant for the victory of epidemic fighting. Moreover, scholars have explored many dimensions related to epidemic prevention and control, such as measures that lead citizens to reduce their frequency of going outside and wear masks during outings [21,22] and ways in which the governments can increase the credibility of information [23]. However, studies to date have not considered the role played by public servants in the prevention and control of the epidemic situation. Considering the scarcity of literature about the behaviors of public servants during the pandemic, this study aims at addressing this knowledge gap by assessing what factors have affected the pandemic prevention behaviors of public servants during the epidemic period. Studying public servants’ responses during a pandemic is helpful to know what measures might encourage public servants to implement behaviors to prevent the spread of an epidemic.

The paper is structured as follows: Section 2 introduces the study’s conceptual model. Section 3 introduces the data collection and processing methods. Section 4 and Section 5 describe the data used and the empirical results, respectively. Finally, Section 6 presents the study’s conclusions.

## 2. Conceptual Model and Hypotheses

**Figure 1:**
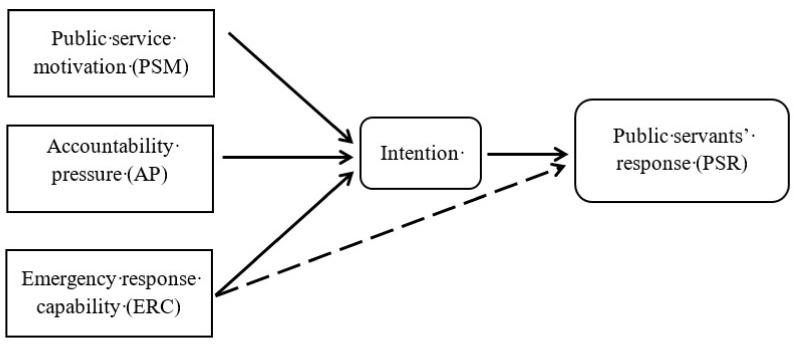
Conceptual model.

The theory of planned behavior (TPB) is frequently used to explain different kinds of behavior in different areas of social sciences [24]. It is an extension of the theory of reasoned action (TRA) [25]. The theory holds that an individual’s behavior largely depends on the intention to perform the behavior; the stronger the intention to engage in a behavior is, the more likely it is to be performed [26]. The intention, in turn, is influenced by three factors: attitudes toward the behavior (ATT), subjective norms with respect to the behavior (SN), and perceived behavioral control (PBC) [27]. At the beginning of its theoretical development, studies on TPB mainly concentrated on health-related behaviors, such as healthy eating behaviors [28], safe sexual behaviors [29], and pro-environmental behaviors [30,31]. TPB has been widely applied in social and behavioral studies to address counterproductive work behaviors [32], higher education learning [33], and customer purchase behaviors [34,35]. Past studies have positioned TPB as a cognitive theory that provides a useful framework for predicting and identifying behaviors, with a high degree of accuracy in predicting behavioral intentions. Based on existing research, we applied TPB to analyze the factors impacting pandemic preventative behavior of public servants during the COVID-19 outbreak. This study used public service motivation (PSM), accountability pressure (AP), and emergency response capability (ERC) as independent variables. We hypothesized that all three variables serve as distinctive and significant predictors of public servants’ behaviors in responding to the pandemic (Figure 1).

### 2.1. Public Service Motivation

Public service motivation (PSM) is a multidimensional concept used to describe the motivators encouraging pro-social behavior [36]. Perry described PSM as a predisposition of individuals to serve the public interest [37] and as a deeply embedded personality trait of individuals who are willing to engage in sacrificial behavior for the good of citizens without reciprocal benefits for themselves [38]. This motive has rational, normative, and affective bases [39]. Past research has indicated that the public servants have a high PSM, as public organizations are more likely to provide opportunities to participate in public service and provide due diligence for individuals [40].

According to the TPB, an individual’s attitude towards behavior refers to the degree to which a person has a favorable or unfavorable evaluation or appraisal of the behavior in question [26]. The evaluation mainly depends on whether the behavior will lead to particular outcomes and whether these outcomes are desirable. Thus, PSM can be regarded as a civil servant’s ATT towards the adoption of public servants’ response to protect the public interest. Scholars have concluded that public servants with higher levels of PSM perform better compared to those with low levels of PSM. This may be because they find their work important and meaningful and are more likely to invest their resources in public service work, keeping them engaged [38,41]. In view of these PSM-related traits, we hypothesized the following:

**Hypothesis** **1.** *PSM is positively related to public servants’ pandemic response.*

### 2.2. Accountability Pressure

Accountability is a relationship between an actor and a forum: the actor has an obligation to explain and to justify his or her conduct; the forum can pose questions and pass judgment, and the actor may face consequences [42]. The actor can be an individual, such as an official or civil servant, or an organization, such as a public institution or an agency. The accountability forum can be a specific person, such as a superior, minister, or journalist, or it can be an institution, such as parliament, court, or audit office [43]. Accountability is widely seen as a tool used by citizens to compel those vested with public power to speak the truth [42,43]. In addition, scholars have conducted detailed research on how to use accountability pressure to improve employee productivity and made many recommendations [44,45].

SN relates to perceived social pressure from family, friends, colleagues at work, and other agents; this pressure causes people to perform or not perform a behavior [26]. During the epidemic, the social pressure faced by public servants was mainly from accountability pressure due to the perception of the epidemic response as inadequate. This study used accountability pressure (AP) to express the perceived SN of public servants. Based on this, we hypothesized the following:

**Hypothesis** **2.** *AP is positively related to public servants’ pandemic response.*

### 2.3. Emergency Response Capability

Emergency response capability (ERC) reflects a comprehensive capability to address such events as natural disasters, sudden public health or safety incidents, or military conflict [46]. ERC is a major indicator assessing the degree of difficulty faced by public servants when preventing epidemics. Scholars have found that the corresponding training for ERC can significantly improve emergency response abilities in an emergency [47,48].

PBC also plays a significant part in the theory of planned behavior. It refers to the perceived ease or difficulty of performing a behavior, and it is assumed to reflect past experience and anticipated impediments and obstacles [26]. PBC influences behavior indirectly by influencing behavior intention, and it directly affects behavior achievement [26,49]. This study used ERC to indicate PBC. The framework for ERC generally consists of three levels: systems, organizational, and individual [50]. This study evaluated ERC at the individual level, focusing on the civil servant. Based on this, we hypothesized the following:

**Hypothesis** **3.** *ERC is positively related to public servants’ pandemic response.*

The theory of planned behavior holds that when a behavior is evaluated more favorably (PSM) and a person feels greater social pressure (AP), in conjunction with a sense of PBC, the behavioral intention is expected to be stronger [51].

### 2.4. Control Variables

Other factors may affect the pandemic preventative behavior of public servants. For example, gender may be one factor leading to differences in behavior of public servants when facing a public emergency. Many scholars have conducted related research. For example, Perry found that men scored higher on the structure of public interest than women in 1997 [52]; in contrast, some scholars have proposed a dimension of compassion for the PSM, in which women consistently score higher than men [37]. As such, the effect of gender may be positive or negative. As people grow older, they generally become more concerned with positively contributing to society, suggesting that age may also impact pandemic preventative behavior [53]. Previous studies found that information diffusion significantly influences the epidemic spread [54]; people with higher levels of education may receive more information about an epidemic than less educated people [55]. Meanwhile, individuals with higher education will thus express higher levels of PSM, because they have internalized the values and norms of public service, such as the importance of contributing, by means of socialization, social identification, and social learning [56]. As a result, they are more likely to take actions that support epidemic prevention and control.

Political countenance (or affiliation) (Poc) is also a factor that may cause differences in the behavior of public servants when facing a pandemic risk. In China, for example, Communist Party members must implement their original intent and oath to “be ready to sacrifice everything for the party and the people” with their actions, especially during the epidemic. The higher the administrative level (Adlevel) of public servants, the more responsibility they bear. As a result, they may face greater stress, driving them to take epidemic prevention more actively and seriously. Meanwhile, some scholars believe that job stress that can be classified as hindrance stress, such as role ambiguity and role conflict, negatively affects job performance, while challenge stress such as workload and job responsibility inversely affects job performance and work engagement [57]. Thus, whether the impact of Adlevel is positive or negative is uncertain. Leaders, as formal authority figures, play an important role in the organization. Studies have shown that obviously displaying pro-social and selfless behavior can prompt observers to also act with kindness and generosity [58]. In this way, leaders can function as models and motivate people to put their values into action [59]. This means public servants in leading positions are more likely to actively participate in epidemic prevention. The length of service (Length) may also impact the selection of public servants for epidemic prevention, as previous studies have demonstrated that as the length of service increases, people feel more exhausted [60] and the tendency to engage in pro-social behaviors declines significantly [61].

## 3. Methods

### 3.1. Ethical Consideration

The study was approved by the School of Economics and Management, Fuzhou University (ED-FZU-SEM-2020010). All the participants submitted informed consent before completing the questionnaire.

### 3.2. Data Collection

All data for this study were collected using an online questionnaire, administered from 5 to 10 February 2020. The survey included the following steps. First, based on the COVID-19 situation, the online questionnaire was designed to explored epidemic awareness and prevention behaviors taken by Chinese public servants. Second, the online questionnaire was then sent by Credamo (a platform for conducting questionnaire surveys) to Master of Public Administration (MPA) students in universities of China. These students, who are working in the government, are participating in the MPA program to be equipped with proper political and ideological qualities and professional ethics and master systematic public management theories, knowledge, and methods. The MPA students were asked to send the online questionnaire to their colleagues, who were also asked to send it to their colleagues. In this way, sufficient sample data could be obtained from the MPA students’ relational networks. Each participant submitted informed consent before completing the questionnaire. Third, a total of 1371 valid questionnaires were retrieved. To ensure data reliability, the data were further cleansed, and some responses were excluded due to a lack of value. The final number of analyzed responses was 1293 respondents, which was a net response rate of 94.31%.

### 3.3. Definition and Measurement of Explanatory Variables

All variables were measured using the online questionnaire; the variables and items are listed in Table 1, and all the items came from the international authoritative measurement scale. For instance, the question of PSM overlapped with a part of Perry’s measurement scale of public service motivation. Most of the variables were quantitative variables. For example, we divided age into different stages, and there was continuity between each stage. The number “1” represents 1–5 years old, and “2” represents 6–10 years old; i.e., the larger the number is, the older the participant is. In addition to the control variables, the variables PSR, PSM, AP, and ERC were described using three questions, and each variable was obtained by means of the aggregation of each of its indicators; each item was answered using a five-point system, ranging from 1 = “strongly disagree” to 5 = “strongly agree”. For example, AP was divided into three kinds of pressure: pressure from superior leaders, pressure from laws and regulations, and pressure from public opinion and social media. The Cronbach’s alpha was 0.7690.

### 3.4. Data Analysis

All data were analyzed using the statistical software Stata/MP, version 14.0 (StataCorp LP., Texas City, USA). The main analysis steps were as follows: First, we generated descriptive statistics of the variables. Second, a correlation matrix was developed to examine the relationships between 10 quantitative variables. Third, we adopted multiple linear regression models to explore the effects of PSM, AP, and ERC on public servants’ response. Finally, a quantile regression was used to assess whether the effects of PSM, AP, and ERC on public servants’ response exhibited group heterogeneity. This study selected the most common threshold of P, i.e., *p* < 0.05, and all of our regression models passed the significance test.

## 4. Results

### 4.1. Descriptive Information

The descriptive statistics of the dependent and independent variables are shown in Table 2. There were 1293 participants in the study. Most participants had a college education or above (63.0%), and there were more males (50.27%) than females (49.73%). With respect to age, 45.86% of respondents were between 20 and 30 years old, and 11.52% were 40–50 years old. In terms of political party, 76.95% of the participants were members of the Communist Party of China; the remaining 23.05% were non-party members and were associated with the Democratic Party, the Communist Youth League, and others. With respect to administrative level, 50.04% of participants were staff members; the lowest number of subjects were at the bureau level, accounting for only 0.77% of the total. Of all the participants, only 21.27% reported holding leadership positions. Finally, 69.37% of the participants reported serving as public servants for less than 10 years.

Before conducting the formal linear regression, a correlation matrix was established to test the correlation between variables. A significant association was observed for 8 of the 10 indicators related to public servants’ response. Table 3 shows the results. Among them, Poc, Leader, and Adlevel were significantly negatively correlated with public servants’ response. Meanwhile, PSM, AP, ERC, and Length were significantly positively related to public servants’ response. Most of the correlation results are consistent with our hypothesis. It is surprising that the dimensions of education and gender did not pass the significance test.

### 4.2. Overall Effect and Stratification Difference

To assess the overall effect, this study employed Model 1 with public servants’ pandemic response (PSR) as the dependent variable and demographic variables as control variables. Next, PSM was added in Model 2. Last, AP and ERC were added to Model 3 and Model 4, respectively.

The results indicate that PSM, AP, and ERC were the key factors influencing public servants’ response (significant at *p* < 0.001) and had different effects. Table 4 shows that AP had the most significant impact on public servants’ response; when AP increased by 1%, the reported willingness of civil servants to actively take actions to prevent the epidemic increased by 0.273%. Hypothesis 2 was supported, as AP was significantly and positively related to public servants’ response. The participants were asked where their accountability pressure mainly comes from when dealing with public emergencies. Of the total subjects, 66.36% said accountability pressure came mainly from superior departments and leaders, and 17.79% reported it coming from public opinion. PSM had the second largest effect on public servants’ response; when PSM increased by 1%, the reported willingness to engage in public servants’ response increased by 0.230%. This supported Hypothesis 1. Finally, when ERC increased by 1%, the reported willingness of public servants to engage in pandemic preparation increased by 0.135%, verifying Hypothesis 3.

For the control variables in Models 1 and 4, while the significance levels differed, it can be seen that the coefficients of gender on public servants’ response were significant and positive. This indicates that after the outbreak, female public servants were more likely to adopt positive preventive actions than males. The administrative level had a significant negative effect on public servants’ response in Models 1 and 3. This indicates that the higher a civil servant’s position was, the more they reported engaging in relevant preventive work. This may explain some differences in the evaluation of “public interest commitment” and “policymaking attractiveness” between street-level bureaucrats (street-level bureaucrats are public employees who interact directly with individual citizens and have substantial discretion in allocating facilities or imposing sanctions) and upper-level management (upper-level managers are individuals who are responsible for making the primary decisions) [62]. Leadership positions also had a negative impact on public servants’ response in Models 2–4, indicating public servants in leadership positions are more likely to participate in epidemic prevention and control. This result is consistent with the impact of administrative level on public servants’ response.

Not all public servants possess the same level of PSR. To ensure the robustness of our results, based on the quantile regression of the data on the 1293 study subjects, this study further verified whether there were differences in the impacts of PSM, AP, and ERC on different quantiles of public servants’ response. A greater number of quantiles is associated with a greater clarity in conditional distributions. This study selected five representative quantiles for analysis: low behavior level group (IVQR_10), medium and low behavior level group (IVQR_25), medium behavior level group (IVQR_50), medium and high behavior level group (IVQR_75), and high behavior level group (IVQR_90).

**Figure 2:**
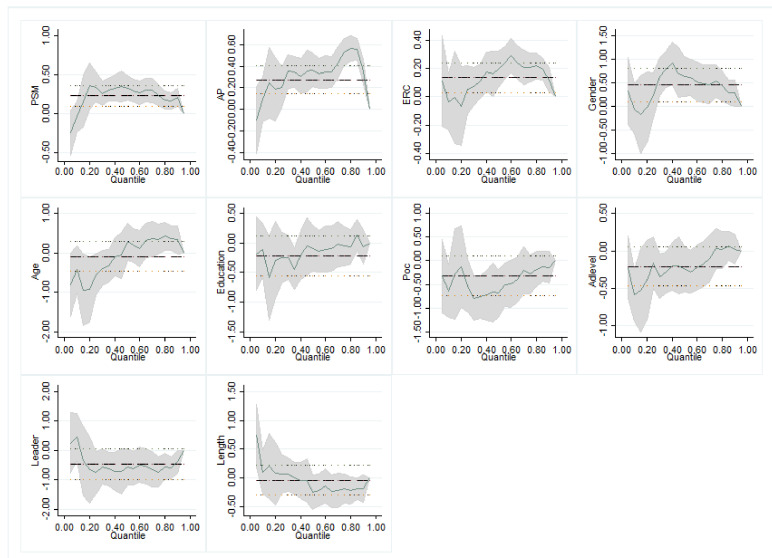
Quantile regression coefficient trends of explanatory variables.

The results of the quantile regression are shown in Table 5 and Figure 2. The table shows that PSM, AP, and ERC had significant heterogeneity effects on public servants’ response. First, with the exception of IVQR_10, the coefficients of PSM of the remaining four quantiles were all significantly positive, with elastic coefficients as high as 0.341, 0.330, 0.236, and 0.208 for IVQR_25, IVQR_50, IVQR_75, and IVQR_90, respectively. This indicates that PSM was reported to significantly improve public servants’ response for most public servants but was not significant for the public servants reporting low levels of these behaviors.

Second, with respect to AP and ERC, the coefficients of IVQR_50, IVQR_75, and IVQR_90 were all significantly positive. AP was more elastic to public servants’ response than ERC. In addition, the improvements in AP and ERC were associated with higher public servants’ response for the IVQR_50 group. The regression coefficient exhibited an inverted U shape as the quantile number increased (see Figure 2). This illustrates that improvements in the public servants’ response first increased and then decreased in response to AP and ERC.

Third, from the low quantile to the high quantile, while the elasticity fluctuated slightly, there was an overall upward trend, with the PSM, AP, and ERC coefficients generally increasing. This indicates that these three variables were generally reported to improve the pandemic prevention performance of public servants. However, the increase in these behaviors was more reflected in IVQR_50, IVQR_75, and IVQR_90. This indicates there were limitations in the degree of the positive impact that PSM, AP, and ERC had on the public servants’ response.

Finally, compared to the overall effect of Model 4 in Table 4, the coefficients of PSM, AP, and ERC in IVQR_50 and IVQR_75 in Table 5 were generally higher. For example, the coefficients of AP in IVQR_50 and IVQR_75 were 0.366 and 0.533, respectively, which were higher than the coefficient of 0.273 in Table 4.

### 4.3. Factors Influencing Pandemic Prevention Performance in Different Job Categories

The differences in job categories determine the different work responsibilities of public servants, which may affect their willingness to engage in pandemic prevention measures when facing the pandemic. Thus, we performed regression on pandemic prevention performance in different job categories.

The coefficients and the significance levels of PSM, AP, and ERC in Table 6 are different in every model, which illustrates that there are indeed some status differences. The variables (including AP, ERC, gender, and leader) have an important influence on the pandemic prevention performance of public servants in integrated management. As for administrative law enforcement, also known as “street-level bureaucrats”, AP, ERC, education, and political countenance have significant effects on pandemic prevention performance. However, for public servants in professional technology, only PSM and AP have a significant influence on pandemic prevention performance.

The coefficients of PSM, AP, and ERC in Table 6 were different from the overall effect in Table 4. Specifically, for professional and technical public servants, when PSM increased by 1%, the reported willingness to engage in public servants’ response increased by 0.627%, which was much higher than 0.230% in Table 4; when AP increased by 1%, the reported willingness of civil servant to actively take actions to prevent the epidemic increased by 0.434%, also higher than the overall effect of 0.273%. As for administrative law enforcement, the coefficient of ERC was 0.299, higher than 0.135 in Table 4.

## 5. Discussion

The goal of this paper was to analyze the effects of PSM, AP, and ERC on public servants’ response when public servants face a pandemic emergency. To accomplish this goal, we established the measurement scale, built a theoretical model based on TPB theory, and applied regression methods to analyze the data collected from Chinese study subjects.

### 5.1. The Factors Contributing to Public Servants’ Response

We ran multiple linear regression models to test the efficiency of the conceptual model that we built based on the TPB. The results confirm that AP, PSM, and ERC all passed the significance test, indicating they were all reported to play a positive role in improving public servants’ response. Among the three independent variables, AP had the greatest impact on public servants’ response, followed by PSM and then ERC. This differed from the previous studies, which concluded that subjective norms were weaker in forming intention than the attitudes toward the behavior [63].

The possible explanations for the positive role that AP can play in increasing public servants’ response are as follows: Accountability for performance has replaced more traditional notions of bureaucratic accountability for both fairness and financial reasons and has become a familiar type of accountability [64]. This shift has provided new criteria for administrative success and what it means to be accountable as a civil servant. As a result, following proper “procedure” is no longer considered a sufficient action by public servants if the result is an inadequate policy solution [65]. Since the 18th CPC National Congress, China has stressed the need to accelerate the transformation of government functions and innovation management methods. Therefore, increased attention has been paid to evaluating government performance, especially the performance of public servants. This performance is closely related to rewards or penalties. Thus, when facing a high degree of accountability pressure (AP), public servants will be more active in epidemic prevention and control to avoid any penalties.

Previous studies have confirmed a strong link between individual PSM and performance in public sector organizations [66,67,68]. Furthermore, the positive effect of PSM on public servants’ response is largely due to the deep personality trait held by individuals who are willing to engage in self-sacrificing behavior for the benefit of their citizens, rather than seeking mutual benefit for themselves [38]. Therefore, public servants with a high degree of PSM are more likely to actively carry out the tasks of epidemic prevention and control to preserve the safety of other people’s lives and property.

ERC had a positive impact on the behaviors of public servants during the epidemic. ERC is the ability to effectively address emergencies, which depends on individual perception of the environment, skills, time, and cost. Public servants having a strong ERC reported believing that the epidemic is highly controllable and expressed more confidence in addressing emergencies. This makes them more inclined to exhibit positive epidemic prevention behaviors.

Moreover, the results of quantile regression show that the effects of PSM, AP, and ERC on public servants’ response were heterogeneous between groups, which means PSM, AP, and ERC failed to positively impact the behavior of all public servants. More specifically, they had little effect on public servants that reported exhibiting low levels of public servants’ response. Instead, the three factors had the greatest impact on public servants reporting medium levels of the desired behaviors. This may because public servants reporting low levels of the public servants’ response may have been ensuring their own safety or taking better care of their families and considered these more important than the public interest. Even though they face greater accountability pressure (AP) or have stronger emergency response capabilities (ERC), not all respondents reported actively participating in epidemic prevention and control during the outbreak.

In addition, we found that the job categories of public servants might also be a factor related to pandemic prevention performance. Among them, PSM and AP have the strongest effects on public servants in professional technology, and ERC has the greatest influence on administrative law enforcement. One possible explanation is the different job types have different effects on performance perceptions and expectations [69], risk perception [70], and job satisfaction [71]. Thus, if we want to improve the specific behaviors of public servants, we cannot make sweeping generalizations, but rather, we need to take targeted incentive measures according to their actual situation.

### 5.2. Policy Recommendations for the Government

When the epidemic broke out, public servants may have faced a conflict between positively preventing the epidemic to safeguard public health and safety and passively avoiding responsibility to protect their own interests. Public servants’ response was essential for controlling the epidemic situation. The theory of planned behaviors provides a new perspective and method for studying behavior in the field of public management, which helps us to better understand the factors contributing to public servants’ behaviors. The more we know about any particular behavior, the more we can influence and change it. Therefore, after considering the roles of the factors influencing public servants’ participation in epidemic prevention and control, the following policy implications can be proposed:

For public servants reporting below a medium level of public servants’ response, measures need to be taken to maximize the factors that maintain or enhance PSM and mitigate the factors that reduce it [41]. This would help public servants actively participate in epidemic prevention and control. Examples would include building trust in the workplace, as creating a strong tie between workplace trust and PSM acts as a catalyst that enables public managers to transform their service propensity into real actions [72]. In addition, transformational leaders who provide a vision, set a positive example, encourage innovation, and cultivate organizational pride can also promote public service motivation [73]. Reducing red tape, implementing reforms, clarifying goals, and empowering employees can also positively impact the PSM of public servants [74].

For public servants reporting medium or higher level of public servants’ response, appropriate material incentives and welfare guarantees may enable public servants to worry less when engaging in relevant epidemic prevention work. In addition to a basic salary, increasing the proportion of incentive and special post allowances can help optimize the salary structure. Further, enhancing employee prospects for career growth [75], with a scientific and feasible promotion mechanism, may help public servants improve their enthusiasm for work. This may make them more proactive in performing related epidemic prevention tasks.

It is important to establish an effective performance evaluation mechanism and accountability system and increase the role of accountability pressure (AP) in promoting the legitimacy of civil servant behavior. Previous studies have shown that performance appraisals influence the expectations made of public servants, influencing their work motivation [76]. In this study, AP was reported to mainly come from superior departments and leaders, and it had the greatest effect on the public servants’ response. Motivating public servants to perform their duties actively through a strict accountability system and mechanism, with some error tolerance, can help distinguish the types of decision-making errors that are tolerated or penalized. This can encourage public servants to take proactive actions in the threat of an incoming public health crisis [77].

It is critical to establish an emergency training system to improve the emergency response capacity (ERC) of public servants. This should strengthen the study of related theories. For example, the government could leverage administrative schools in different universities to regularly conduct theoretical training courses for public servants, including methods for interpreting different types of frequent emergencies. This would also enhance ERC from a practical level. Theory-based training should be followed with different simulation exercises for different types of public emergencies, to increase civil servant familiarity with emergency plans and to improve the efficient use of emergency plans in times of crisis.

Furthermore, in order to better improve the enthusiasm of public servants for epidemic prevention, targeted measures can be taken for public servants of different job categories. For public servants in professional technology, measures should be taken to improve their PSM and AP from internal and external aspects, respectively. As for comprehensive management, increasing the pressure of accountability can effectively improve the public servants’ response. With respect to public servants in administrative law enforcement, the key is to improve their ERC through theoretical teaching and practical training.

### 5.3. Limitations

First, due to limited time and cost, the sample size for the questionnaire was small. This may lead to a slight deviation between the actual situation and the calculation results. Second, the sample data were optimized and screened; however, because the study data were mainly obtained through participants’ self-evaluation, participants may have concealed the actual situation. In the future, the data should be optimized in a variety of ways.

## 6. Conclusions

Based on questionnaire data collected from 1293 public servants in China, our research found that PSM, AP, and ERC significantly impacted the epidemic prevention behaviors of public servants exhibiting a medium level of the behavior or higher. AP was found to play the most important role, providing useful information about how to mobilize the enthusiasm of public servants in the face of public security emergencies. The level of epidemic prevention behaviors reported by public servants was also related to job category, gender, education, and administrative level. The main innovation of this research is that it is the first known study to focus on the epidemic prevention and control behaviors of public servants and to discuss the main factors influencing these behaviors. Moreover, this study is one of the first to apply TPB to understand the behavior of public servants in public management settings. This broadens the application scope of TPB theory and provides a new perspective and method for behavioral studies in the field of public management.

## Figures and Tables

**Table 1:** Variable description.

Variable	Indicator	Variable Description
PSR	“As long as the superior issues a prevention and control task, I can deploy and implement the prevention and control work vigorously”	(1) Strongly disagree, (2) Disagree, (3) It does not matter, (4) Agree, (5) Strongly agree
	“My execution ability is very strong, and I can effectively implement and execute superior prevention and control instructions”	
	“Without the urging of the superior, I can still actively complete the prevention and control tasks assigned by the superior”	
PSM	“I think participation in public service is the duty of every citizen”	(1) Strongly disagree, (2) Disagree, (3) It does not matter, (4) Agree, (5) Strongly agree
	“It’s important for me to serve the public”	
	“I am willing to make great sacrifices for the overall interests of society”	
AP	“I strictly abide by laws, rules, and procedures to carry out my work”	(1) Strongly disagree, (2) Disagree, (3) It does not matter, (4) Agree, (5) Strongly agree
	“I strictly implement the principles and methods of higher authorities’ instructions”	
	“In response to this epidemic, we attach great importance to publicity work for the media and society”	
ERC	“No matter how much disaster information I receive, I can grasp the key information points”	(1) Strongly disagree, (2) Disagree, (3) It does not matter, (4) Agree, (5) Strongly agree
	“Even if I haven’t got enough information, I can make quick response decisions”	
	“When I receive the arrangements for epidemic prevention and control, I can quickly put aside my work or life arrangements to respond”	
Gender	“What is your gender?”	(1) Male, (2) Female
Age	“How old are you?”	(1) 20–30, (2) 31–40, (3) 41–50,(4) Older than 50
Education	“What is your highest education level (including your current level of study)?”	(1) High school, (2) College, (3) Undergraduate, (4) Graduate and above
Poc	“Are you a party member?”	(1) Yes, (2) No
Adlevel	“What is your administrative level?”	(1) Administrative level, (2) County level, (3) Township level, (4) Staff member, (5) Clerk
Leader	“Are you in a leading position?”	(1) Yes, (2) No
Length	“How long have you worked?”	(1) 1–5, (2) 6–10, (3) 11–15, (4) 16–20, (5) More than 20 years
Job category	“What is your job category?”	(1) Integrated management, (2) Administrative law enforcement, (3) Professional technology, (4) Other

**Table 2:** Descriptive statistics of the variables (*n* = 1293).

Variable	Categories	Mean ± SD/Total No.	Range/Percent
Gender	Males	650	50.27%
	Females	643	49.73%
Age	20–30	593	45.86%
	31–40	511	39.52%
	41–50	149	11.52%
	Older than 50	40	3.09%
Education	High school	1	0.08%
	College	22	1.70%
	Undergraduate	455	35.19%
	Graduate and above	815	63.03%
Poc	Party member	995	76.95%
	Non-party member	298	23.05%
Adlevel	Administrative level	10	0.77%
	County level	144	11.14%
	Township level	357	27.61%
	Staff member	647	50.04%
	Clerk	135	10.44%
Leader	Leader	275	21.27%
	Non-leader	1018	78.73%
Length	1–5	428	33.10%
	6–10	469	36.27%
	11–15	182	14.08%
	16–20	75	5.80%
	More than 20 years	139	10.75%
Job category	Integrated management	851	65.82%
	Administrative law enforcement	214	16.55%
	professional technology	208	16.08%
	Other	20	1.55%
PSR	-	10.60 ± 3.214	[3,15]
PSM	-	13.56 ± 1.469	[3,15]
AP	-	13.42 ± 1.509	[3,15]
ERC	-	12.14 ± 1.871	[3,15]

**Table 3:** Correlation matrix of the studied variables.

Variables	PSR	PSM	AP	ERC	Gender	Age	Education	Poc	Adlevel	Leader	Length
PSR	1										
PSM	0.193 ***	1									
AP	0.203 ***	0.425 ***	1								
ERC	0.176 ***	0.411 ***	0.382 ***	1							
Gender	−0.023	−0.097 ***	−0.048 *	−0.135 ***	1						
Age	0.057 **	0.139 ***	0.083 ***	0.240 ***	−0.212 ***	1					
Education	−0.037	−0.061 **	−0.018	−0.103 ***	0.002	−0.094 ***	1				
Poc	−0.062 **	−0.067 **	−0.035	−0.053 *	0.120 ***	−0.180 ***	−0.155 ***	1			
Adlevel	−0.092 ***	−0.105 ***	−0.046 *	−0.179 ***	0.247 ***	−0.468 ***	−0.041	0.228 ***	1		
Leader	−0.086 ***	−0.046 *	−0.028	−0.115 ***	0.192 ***	−0.417 ***	−0.053 *	0.208 ***	0.559 ***	1	
Length	0.073 ***	−0.184 ***	0.122 ***	0.231 ***	−0.231 ***	0.828 ***	−0.082 ***	−0.230 ***	−0.510 ***	−0.470 ***	1

Standard errors in parentheses; * *p* < 0.05, ** *p* < 0.01, *** *p* < 0.001.

**Table 4:** The multiple linear regression models (*n* = 1293).

Variables	Model 1	Model 2	Model 3	Model 4
	PSR	PSR	PSR	PSR
Gender	0.364 *	0.434 **	0.435 **	0.462 **
	1.930	2.330	2.360	2.510
Age	−0.094	−0.055	−0.035	−0.086
	−0.440	−0.250	−0.160	−0.380
Education	−0.296 *	−0.237	−0.249	−0.214
	−1.720	−1.390	−1.450	−1.260
Poc	−0.363	−0.315	−0.318	−0.322
	−1.610	−1.440	−1.470	−1.490
Adlevel	−0.239 *	−0.211	−0.224 *	−0.202
	−1.760	−1.570	−1.700	−1.520
Leader	−0.366	−0.470 *	−0.477 *	−0.477 *
	−1.330	−1.730	−1.760	−1.770
Length	0.093	−0.009	−0.042	−0.035
	0.680	−0.060	−0.290	−0.240
PSM		0.412 ***	0.277 ***	0.230 ***
		6.830	4.320	3.310
AP			0.315 ***	0.273 ***
			4.700	3.870
ERC				0.135 **
				2.160
Constant	13.034 ***	7.322 ***	5.066 ***	4.454 ***
	12.320	5.560	3.460	3.050
Observations	1293	1293	1293	1293
R-squared	0.017	0.051	0.069	0.074
adj_R2	0.066	0.066	0.066	0.066
F	9.129	9.129	9.129	9.129

Standard errors in parentheses; * *p* < 0.05, ** *p* < 0.01, *** *p* < 0.001.

**Table 5:** The quantile regression model (*n* = 1293).

Variables	Model 1	Model 2	Model 3	Model 4	Model 5
	IVQR_10	IVQR_25	IVQR_50	IVQR_75	IVQR_90
PSM	−0.040	0.341 *	0.330 ***	0.236 ***	0.208 *
	−0.280	2.560	3.970	3.510	2.230
AP	0.093	0.208	0.366 ***	0.533 ***	0.351 ***
	0.690	1.630	4.620	8.280	3.920
ERC	−0.356	0.052	0.194 **	0.206 ***	0.119 *
	−0.320	0.500	2.980	3.900	1.620
Gender	−0.049	0.243	0.634 **	0.548 **	0.286
	−0.130	0.690	2.900	3.090	1.160
Age	−0.409	−0.549	0.285	0.347	0.333
	−1.00	−1.420	1.180	1.780	1.230
Education	−0.111	−0.243	−0.087	−0.050	−0.066
	−0.320	−0.740	−0.430	−0.300	−0.280
Poc	−0.640	−0.520	−0.690 **	−0.286	−0.137
	−1.430	−1.240	−2.630	−1.350	−0.460
Adlevel	−0.587 *	−0.156	−0.234	0.035	0.024
	−2.140	−0.600	−1.430	0.270	0.130
Leader	0.458	−0.751	−0.558	−0.729 **	−0.387
	0.840	−1.460	−1.740	−2.800	−1.070
Length	0.098 *	0.069	−0.242	−0.181	−0.185
	0.370	0.280	−1.550	−1.430	−1.050
_cons	8.618 **	4.665	1.507	0.789	5.649 **
	2.940	1.690	0.880	0.570	2.920

Standard errors in parentheses; * *p* < 0.05, ** *p* < 0.01, *** *p* < 0.001.

**Table 6:** Regression on pandemic prevention performance in the different job categories.

Variables	Integrated Management	Administrative Law Enforcement	Professional Technology	Other
PSM	0.108	0.172	0.627 ***	−1.184
	1.240	1.100	3.880	−1.090
AP	0.203 *	0.315 *	0.434 **	0.511
	2.400	2.110	2.820	0.740
ERC	0.193 **	0.299 *	−0.007	0.836
	2.760	2.310	−0.060	1.070
Gender	0.799 ***	−0.620	−0.333	−0210
	3.500	−0.140	−0.770	−0.110
Age	0.196	−0.102	−0.623	−2.303
	0.780	−0.210	−1.430	−0.820
Education	−0.060	−0.858 *	−0.229	−1.824
	−0.280	−2.210	−0.530	−0.850
Poc	0.101	−1.025 *	−0.102	−1.334
	0.360	−1.980	−0.220	0.420
Adlevel	−0.106	0.031	−0.301	−0.951
	−0.590	0.100	−1.120	−0.630
Leader	−0.948 **	0.843	1.034	−1.807
	−2.970	1.330	1.410	−0.400
Length	−0.112	0.328	0.053	1.081
	−0.680	1.010	0.190	0.620
Constant	5.706 **	2.808	5.066 ***	26.753
	2.800	0.850	3.460	1.620
Observations	851	214	208	20
R-squared	0.065	0.166	0.214	0.417
adj_R2	0.054	0.124	0.174	−0.232
F	5.880	4.030	5.360	0.640

Standard errors in parentheses; * *p* < 0.05, ** *p* < 0.01, *** *p* < 0.001.

## Data Availability

The data presented in this study are available on request from the corresponding author. The data are not publicly available due to the data also forms part of an ongoing study.
